# Hesitations and relative proeminence in prosodic constituents in children's speech

**DOI:** 10.1590/2317-1782/20212020220

**Published:** 2022-01-10

**Authors:** Cristyane de Camargo Sampaio Villega, Lourenço Chacon

**Affiliations:** 1 Programa de Pós-Graduação em Estudos Linguísticos, Universidade Estadual Paulista – UNESP - São José do Rio Preto (SP), Brasil.

**Keywords:** Child Language, Linguistics, Speech Therapy, Communication, Language Development, Speaks

## Abstract

**Purpose:**

to verify if hesitations would occur, preferably, in strong or weak positions of four of the prosodic constituents: phonological utterance, intonational phrase, phonological phrase and clitic group.

**Methods:**

the data were extracted from a bank composed of 147 interview situations recorded with children aged 5-6 years. Was used the principle of relative prominence for the analysis of prosodic constituents. From this principle, the hesitant occurrences identified in prominent elements in the organization of each of the prosodic constituents was considered as for strong position and, as in a weak position, the hesitant occurrences identified in parts of constituents that surround the prominent positions. The judges detected 2.399 hesitant occurrences.

**Results:**

the following total hesitations were identified in strong and weak positions, respectively: (1) in the phonological utterance = 305 (28.37%) and 770 (71.63%); (2) in the intonational phrase = 285 (20.67%) and 1094 (79.33%); (3) in the phonological phrase = 129 (16.49%) and 653 (83.51%); and (4) in the clitic group = 154 (15.21%) and 859 (84.79%).

**Conclusion:**

although hesitant occurrences have been identified in strong positions in all prosodic constituents analyzed, there was prevalence due to the weak position. This result corroborates studies that claim that hesitations would occur in non-nuclear prosodic portions. Furthermore to this confirmation, the results reinforce the effectiveness of the prosodic phonology model in relation to the principle of relative prominence.

## INTRODUCTION

The literature focusing on hesitations has considered different theoretical and methodological perspectives, often associating hesitation with disfluency by investigating the factors that distinguish them. The search for (dis)fluency standards includes researching the relation between normality and pathology^(1-7)^, situations in which hesitations are regarded as common disfluency marks, that is, marks that break speech flow, considered common to all speakers^(1-7)^.

Diverging from this approach, other studies assume that fluency is abstract and ideal, since in actual communication situations, speech is characterized by the presence of elements that break its flow, in addition to assuming that truly continuous flow is rarely achieved in spontaneous speech. In this respect, studies normally investigate the role of hesitations in: (1) cognitive speech planning^(8-10)^, (2) interactional aspects of spoken text formulation ^(11, 12)^, and (3) subjective and discursive aspects of speech^(13-19)^.

Some of the studies that investigate the role of hesitation without focusing on the fluency/disfluency relation address the relation between hesitation and prosody^(20-25)^. We highlight that the term *prosody* includes several phenomena, such as “[...] parameters of pitch, intensity, duration, pause, speech velocity, as well as the study of systems of pitch, intonation, accent, and rhythm of natural languages”^(26)^. Therefore, there are two points of interest in prosodic studies: the “more phonetic” and “more phonological”^(27)^.

The literature includes studies that associate hesitations with both prosodic aspects regarded as phonetic^(24, 25)^ and prosodic aspects regarded as phonological.

The phonological point of view has focused on investigating child speech^(13, 20)^, and adult speech ^(22)^ and establishing a comparison between them ^(23)^. Despite having analyzed subjects from different age groups, these studies reached a similar conclusion: more fluent parts of the utterance – those without hesitation marks – appeared in accent-bearing units or intonational nuclei, while disfluent parts – marked by hesitations – occurred in peripheral and bordering parts, before the nuclei, that is, “[...] before the constituent prosodic head”^(22)^.

In child speech, such a distribution of hesitations has been interpreted as “[...] unsuccessful attempts at segmentation into prosodic blocks”^(13)^. This arrangement between fluent and disfluent parts in child speech has also been interpreted as “[...] prosodic adjustment, especially of a rhythmic nature, in the elaboration of longer and more discursively complex utterances”^(22)^.

An analysis of hesitation marks in adult speech revealed that the distribution of hesitations was not prosodically random, since they appeared in prosodically weak parts of utterances^(22)^.

In turn, a comparison of adult and child speech shows that hesitations manifest differently in adults and children, presenting a non-random distribution in terms of their relationship with prosodic constituents. As highlighted in previous studies, hesitations often occur together with unstressed syllables at the beginning of utterances but never with accent-bearing syllables. We also found that hesitations showed the “[...] child’s sensitivity to prosodic edges at the same time as in more complex elaborations of the production of longer utterances and the introduction of the subject to more elaborated semantic-pragmatic-discursive constructions”^(23)^.

Although these studies highlighted relevant issues – such as the interaction between hesitation and prosody and the non-random distribution of hesitations, when considering their relation to prosody –, many limitations were observed. For example, the first study mentioned^(13)^ does not specify either the number of children or prosodic constituents analyzed. The second study^(22)^, despite focusing on three constituents of different prosodic dimensions, was conducted with a single adult subject, who was analyzed only for hesitations that linguistically manifested as *hesitation repetition* and *hesitation elongation*. Similarly, the third study^(23)^ also focused on specific hesitation marks (*repetition* and *elongation*) by comparing the speech of only one child and one adult.

Given these limitations, our goal is to verify whether these hesitations would occur in fact in non-prominent positions of four of the constituents in the prosodic hierarchy: *phonological utterance*, *intonational phrase*, *phonological phrase,* and *clitic group*. We chose these four constituents for being those that show the different organizational levels above the word in the speech continuity, that is, different degrees of combinations between words in the utterance production.

Our study is aimed at expanding the range of the above-mentioned studies since our analysis highlights the following elements: (1) a large number of child speech productions, specifically 147 interview situations; (2) events involving all hesitation marks appear in the analyzed data: silent pause, filled pause, hesitation elongation, sudden cut-off, hesitation repetition, stuttering, and false beginning, and (3) four of the constituents that make up the prosodic hierarchy.

We consider that this study can contribute to enlarging the theoretical understanding of the relations between hesitation and prosody in their typical operations in the context of child speech. By expanding such a theoretical perspective, we hope that this research contributes to improved training of language therapists, thereby facilitating the development of new resources for child speech assessment and therapy.

## METHODS

### Ethical procedures

This study was approved by the Research Ethics Committee of the Faculty of Philosophy and Sciences, São Paulo State University “Júlio de Mesquita Filho” – UNESP – Marília, protocol number 0132/2010.

For data collection and the development of this research, we included children whose caregivers previously signed an Informed Consent Form. In addition, all children were subjected to speech screening. Those who presented a deviating pattern after screening were excluded from the sample and forwarded for evaluation.

### Sample

The material analyzed was obtained from the ACoLI database – *Apropriação do Conhecimento na Linguagem Infantil* (Knowledge Appropriation in Child Language), which includes speech samples from 24 children with an average age of 5.7 years old. During the data collection period (2011), these children attended full time the second level of municipal schooling for early childhood education (EMEI) in regional areas of the state of São Paulo.

The collation of the ACoLI database during 2011 was based on ten pedagogical workshops carried out as follows: (i) two documenters videotaped monthly sessions in the classroom guided by the teacher in charge; (ii) one week after recording the workshops, each child in the classroom was interviewed individually by one documenter; (iii) the interviews were fully recorded (audio and video) in an acoustic cabin set up in the institution using high precision equipment.

Subsequently, each interview recording (a total of 147) was transcribed by members of the Research Group “Language Studies” (GPEL/CNPq), specifically trained to realize the transcriptions. We organized the data by identifying the children as follows: S01, S02... S23, S24. The documenters received the identifications D01 and D02. Finally, the interview situations were numbered from 1 to 10 according to the chronology of each pedagogical workshop.

### Analysis criteria

We considered the following linguistic marks for hesitations: silent pause (*+*), filled pause (*éh*, *áh*, *hum*), hesitation elongation (**::**), hesitation repetition (repeated *words*), sudden cut-off (/), stuttering (*part of repeated words*), and false beginning (*beginning of an abandoned utterance*)^(11, 15)^.

We selected the four constituents whose prosodic characteristics were above the level of the word according to Prosodic Phonology ^(28)^ since these involve not only the structural characteristics of individual words, but also the relationship between them – continuity being strongly related to fluency in utterance production. These included the constituents *Phonological utterance*, *Intonational phrase*, *Phonological phrase*, and *Clitic group*. It is worth highlighting that in the model of Prosodic Phonology ^(28)^, these four constituents are hierarchically arranged so that (in descending order) a phonological utterance is composed of one or more intonational phrases, which, in turn, are constituted by one or more phonological phrases, formed by one or more clitic groups ^(28, 29)^.

Within each of the four constituents, the hesitation events were classified as occurring in prosodically strong and weak positions by considering the *Principle 4* ^(28)^ of Prosodic Phonology. This principle describes the relation of a relative prominence established between their parts so that in a given prosodic constituent an element has **strong** **value** while the remaining surrounding elements have **weak value**. Therefore, we defined the positions as follows:

- **strong position** – corresponds to the highlighted element in the arrangement of each of the levels in the following prosodic hierarchy analyzed:
  - prominent *intonational phrase* in a *phonological utterance* formation.
  - prominent *phonological phrase* in an *intonational phrase* formation.
  - prominent *clitic group* in a *phonological phrase* formation.
  - *phonological word* in a *clitic group* formation*.*
- **weak position** – corresponds to constituents and/or parts of constituents surrounding the above-mentioned strong positions.

### Quantification of utterances

Considering all 147 interview situations, we accounted for an average of 260.7 seconds of duration per interview and an average of 33.82 utterances produced by the children per interview. However, we excluded cases without linguistic material, such as utterances in which the child answered using gestures (Example 01) or moments of silence and/or when the interviewer waited for an answer (Example 02):

**Example 01:** (interview situation 01)

***D02*** *eh os outros também tinham né?*^1^

***S01*** *((faz gesto de sim com a cabeça))*^2^

**Example 02:** (interview situation 02)

***D01*** *quem eram as personagens da história?*^3^

***S02*** *((longa pausa - o interlocutor fica à espera de uma resposta))*^4^

A total of 3,674 utterances presented linguistic material, from which, the examiners identified those with and without hesitation events at some point in the linguistic chain, presented in Table 1 below:

**Table 1:** Distribution of utterances with and without hesitation events

**UTTERANCES**	**N. (%)**
**Without hesitation events**	1.880 (53.05%)
**With hesitation events**	1.674 (46.95%)
**Total**	**3.674 (100%)**

**Caption:** N ***=*** total number of utterances

Example of utterance without hesitation event:

**Example 03:** (interview situation 06)

***D01*** *eu lembro que você foi o único na sala que lembrou como é que o Portinari morreu + conta pra mim*^5^

***S04*** *com o chumbo da tinta*^6^

Next, we introduce examples of utterances with hesitation events at some point in the linguistic chain:

**Example 04:** (interview situation 05)

***D02*** *me conta o que que ia de ingrediente nesse bolo?*^7^

**Example 05:** (interview situation 09)

***D01*** *e do que vocês brincaram com elas?*^8^

***S13*** *éh de basquete + de boliche + de:: + de:: ++ de:: ++ batatinha quente*^9^

Example 04 shows only one hesitation event indicated by a complex hesitation mark (*+ hum +*) for combining two silent pauses (+) and a filled pause (*hum*). Example 05, in turn, demonstrates two events: the first is indicated by a simple hesitation mark – a filled pause (*éh*), while the second is indicated using a complex hesitation mark (*de:: + de:: ++ de:: ++*) combining hesitation repetition, hesitation elongation, and silent pause.

As in examples 04 and 05, all utterances in all speech productions containing hesitations had either a single hesitation event, such as in 04, or more than one hesitation event, like in 05. Overall, **2,399 hesitation** **events** were identified.

### Statistical analysis

We performed the statistical treatment of the data on *Statistica* software (version 7.0) by conducting descriptive and inferential analyses. We also used a parametric test of *Student T-test* for dependent variables to compare the hesitation events identified in strong and weak positions in each of the four prosodic constituents. This parametric test was chosen due to its not violating the normality test curve , adopting a value of α ≤ 0.05.

## RESULTS

The examiners identified hesitations at points of the linguistic chain related to the four prosodic constituents analyzed (phonological utterance, intonational phrase, phonological phrase, and clitic group) in all 147 interviews. Table 2 presents the distribution of such hesitations in strong and weak positions in the four prosodic constituents, in addition to their statistical distribution:

**Table 2:** Descriptive and inferential statistical analyses of the distribution between strong and weak positions within the four prosodic constituents

**Prosodic constituents**	**Strong position**	**Weak position**	**Student t Test**
***Phonological utterance*** (*n* = 1,075)	28.37% (305)	71.63% (770)	t = - 4.072 p = 0.0005* df = 22
***Intonational phrase*** (*n* = 1,381)	20.64% (285)	79.36% (1096)	t = - 5.408 p = 0.00002* df = 22
***Phonological phrase*** (*n* = 788)	16.37% (129)	83.63% (659)	t = - 5.444 p = 0.00001* df = 22
***Clitic group*** (*n* = 1,013)	15.20% (167)	84.80% (859)	t = - 7.0441 p = 0.0001* df = 22

T Test for dependent samples (p≤0.05)

**Caption:** *n* ***=*** total number of hesitation events per hierarchy level

* = statistically significant difference

Table 2 shows that the hesitations most frequently appeared in weak positions in the four prosodic constituents.

Below are examples of hesitation events identified in **strong** and **weak** positions in the four constituents analyzed. Firstly, an example of strong position in a phonological utterance:

**Example 06:** (interview situation 10)

***D01*** *você lembra de mais alguma coisa que vai ter na formatura?*^10^

***S12*** *depois que nós parar de dançar + nós vamos beber uma aguinha* ***e vamos + e vamos + e vamos*** *embora*^11^

In example 06, the phonological utterance is composed of three intonational phrases: (1) *depois que nós parar de dançar*; (2) *nós vamos beber uma aguinha*; (3) *e vamos embora*. According to the principles that guide this hierarchy level, the intonational phrase to the right of the utterance is naturally given a strong value to delimit the prosodic and syntactic ends of the utterance. As can be seen, the hesitation event *e vamos + e vamos + e vamos* – indicated by the hesitation repetition and silent pause marks – was observed precisely on the intonational phrase corresponding to the strong position of the utterance.

Below is an example of a hesitation event identified in a weak position of a phonological utterance:

**Example 07:** (interview situation 01)

***D02*** *como é que era o tambor?*^12^

***S15*** ***+ é::*** *só colocar na cintu::ra + e tocar com dois pauzinhos*^13^

The phonological utterance in example 07 is constituted by two intonational phrases: (1) *é só colocar na cintura* and (2) *e tocar com dois pauzinhos.* The hesitation event – indicated by the silent pause and hesitation elongation (*+ é::*) marks – occurred in the first intonational phrase. Given that it precedes the intonational phrase bearing the prosodic accent, the hesitation event is placed in the prosodically weak part of the phonological utterance.

Below is an example of a hesitation event identified in a strong position of the intonational phrase:

**Example 08:** (interview situation 01)

***D01*** *é sobre o que essa coisa super importante que você quer falar?*^14^

***S13*** *eu quero falar do:: ++ xilofone*^15^

In example 08, the intonational phrase *eu quero falar do xilofone* is made up of two phonological phrases: (1) *eu quero falar* and (2) *do xilofone*. As it is an intonational phrase without emphatic processes in any of its phonological phrases, the hesitation event – indicated by the hesitation elongation and silent pause marks (*do:: ++* ) – was identified within the second phonological phrase, which had significant prosodic emphasis, therefore gave a strong value to this intonational phrase. This greater emphasis in the second phonological phrase is reinforced by the fact that it corresponds to the part that ends an utterance.

Below is an example of hesitation events identified in a weak position of an intonational phrase.

**Example 09:** (interview situation 08)

***D02*** *e chegando lá na casa o que que eles fizeram?*^16^

***S20*** *hum:: + o rato da cidade ofereceu + uma:: + mesa com MUIta comida*^17^

Example 09 also contains only one intonational phrase, composed of three phonological phrases: (1) *o rato* *da cidade*; (2) o*fereceu* *uma mesa*; and (3) *com MUIta comida*. In this example, two hesitation events were identified (*hum:: +*, indicated by the filled pause, hesitation elongation, and silent pause marks) and (*+ uma:: +*, indicated by a combination between silent pauses and elongation), both in phonological phrases before the prominent phonological phrase (*com MUIta comida*) of the intonational phrase, thus appearing in the weak parts of the constituent. We highlight that the prosodic prominence in this last phonological phrase is reinforced by the presence of an element transcribed with capital letters (showing emphasis in the speech).

After introducing the examples of hesitation events in intonational phrase constituents, we now describe them in the constituent immediately below: phonological phrase. Below is an example of a hesitation event identified in a strong position:

**Example 10:** (interview situation 07)

***D01*** *quais instrumentos que você lembra?*^18^

***S14*** *aquela que as criancinhas ‘tava* ***to/ + éh tá + to*** *cando*^19^

Example 10 shows an intonational phrase made up of three phonological phrases: (1) *aquela*; (2) *que as criancinhas*; and (3) *tava tocando*. The hesitation event (*to/ + éh tá +*) – shown as a combination of stuttering, silent pause, and filled pause – occurs in the third phonological phrase (*tava tocando*), which, in turn, is composed of two constituents immediately below: clitic groups (1) *tava* and (2) *tocando*. According to the rules of relative prominence in this prosodic hierarchy level, in languages such as Brazilian Portuguese, the clitic group to the right of a phonological phrase is always strong, while all the remaining clitic groups are always weak. The hesitation event in example 10 occurs in the clitic group to the right, thus attributed a strong position.

Below is an example of a hesitation event in a weak position of a phonological phrase:

**Example 11:** (interview situation 09)

***D01*** *e do que vocês brincaram com elas?*^20^

***S13*** *de boliche + de:: + de:: ++ de:: ++ batatinha quente*^21^

In example 11, the hesitation event indicated by the hesitation repetition, hesitation elongation, and silent pause marks (*de:: + de:: ++ de:: ++*) occurs in the phonological phrase *de batatinha quente*, which, in turn, is constituted of clitic groups (1) *de batatinha* and (2) *quente*. The hesitation event appears in the clitic group on the left, with weak value (*de:: + de:: ++ de:: ++ batatinha* ), since in this hierarchy level corresponding to the phonological phrase, the prominent part is always in the clitic group on the right – in this case corresponding to the word *quente*.

Next is an example of a hesitation event in a strong position of the clitic group:

**Example 12:** (interview situation 08)

***D02*** *por que que a gente fica forte e saudável se comer a moringa?*^22^

**S08** *pra* ***gen::te:: +*** *não ficar* doente^23^

According to the principles of Prosodic Phonology, the constituent *clitic group* is formed by a content word and all clitic elements (monosyllabic unaccented words, like articles and some prepositions, conjunctions, and pronouns). Therefore, a strong position occurs in this constituent when the hesitation event is identified in the content word that constitutes it. Example 12 shows that the clitic group *pra gente* is composed of a clitic element *pra* and a content word *gente*. The hesitation event indicated by the hesitation elongation and silent pause marks (*gen::te:: +*) occurred precisely at the content word, therefore, in the strong position of the clitic group.

Below is an example of a hesitation event in a weak position of the clitic group:

**Example 13:** (interview situation 02)

***D02*** *e aí o que elas foram fazer lá*^24^

***S02*** *elas correram* ***na + na*** *descida*^25^

In example 13, the hesitation event *na + na* – indicated by hesitation repetition and silent pause marks – occurred in the unaccented monosyllable of the clitic group *na descida*. This hesitation event is classified as being in the weak position of the clitic group, since according to the relative prominence rule in this prosodic constituent, the element considered clitic is always weak, while the content word is always strong.

Having demonstrated the results and hesitation events for the four prosodic constituents analyzed, we will now discuss our findings.

## DISCUSSION

The results demonstrated that the hesitations were distributed both in strong and weak positions for each of the four prosodic constituents analyzed. However, a higher event percentage occurred for the **weak/non-prominent** position in all constituents. These differences also proved statistically significant. This prevalence of hesitation events in weak positions is in line with studies addressing the relation between hesitations and prosody (from a phonological point of view). According to the literature, the occurrence of hesitations is most frequently identified in non-prominent parts of constituents^(13, 22, 23)^. One of these studies^(22)^ found that hesitations do not tend to occur in prominent sections, but in sections that are “[...] peripheral and at the edge, before the nucleus [prominent part]”^(22)^. In other words, the distribution of hesitations was not prosodically random since it followed a general pattern of prosodic behavior.

Therefore, in our results, hesitation events tend to occur in weak positions – before the relative prominence –, as demonstrated in the following examples: 07, in a phonological utterance; 09, in an intonational phrase; 11, in a phonological phrase, and 13, in a clitic group.

Even though our results were corroborated by the literature, we draw attention to the following statement from a study that addressed the relation between hesitations and prosodic constituents in child speech: “Strictly speaking, **I have never found**, in data of [hesitations], cases of hesitation repetition [...] occurring in phrasal accent-bearing syllables [...]”^(23)^ (the authors of this study highlight).

However, the following situations were found by analyzing the data in this study:

**Example 14:** (interview situation 01)

***D01*** *e o tambor também é feito de corda?*^26^

***S02*** *não + tambor é feito + é fe::ito + com negócio embaixo com pra::to*^27^

In example 14, the hesitation event occurs in the phonological phrase *é feito*, composed of two clitic groups (1) *é* and (2) *feito*. In this event, we identified hesitation repetition in the whole structure, including therefore, in its strong part: the clitic group *feito*. Diverging from the literature^(23)^, events such as in Example 14 show that hesitation repetitions, although to a lesser extent, may occur in the prominent element as well, that is, the element bearing the prosodic accent.

Consequently, even though hesitation events in weak positions have prevailed in all analyzed constituents, a number of the hesitation events also appeared in a strong position. Indeed, in contrast to the findings of studies addressing the relations between hesitation and prosody^(11, 22, 23)^, in addition to Example 14, which shows hesitation in strong position at the phonological phrase level, we also found hesitations in strong positions in the remaining prosodic constituents, as in the following examples: 06, in a phonological utterance, 08, in an intonational phrase, and 12, in a clitic group. Therefore, it can be considered that not only the weak value itself would cause hesitations to emerge, but also that, although to a lesser extent, such an emergence could result (as well) from the prominence relation between the parts of a single constituent – which would exemplify its presence (as well) in prosodically strong parts.

Another finding in the data shows that among the hesitation events in weak positions, the clitic group was the most frequent constituent. The phonological clitic group coincides with the category used in a study^(11)^ which classified the types of words with hesitation events, namely functional items – such as articles, prepositions, and conjunctions. Even though the aforementioned study used a different methodology to ours (it did not relate hesitations to prosodic phonological constituents), in addition to a different population (adult speech), the results converge, since 50% of the hesitations found in adult speech involved the functional items.

In another study^(30)^ with similar results, the authors state that “[...] points of significant choice lead the speaker to have difficulty in selecting the next word in the speech flow”^(30)^. The authors consider that functional words represent a minor formulation difficulty in relation to content words. Therefore, we should consider that in the relative prominence functioning particular to the prosodic hierarchy level, the clitic group stands out in terms of the occurrence of hesitations since it involves not only different prominence degrees (as in constituents above the clitic group) but the absence/presence of prominence itself, as it is about the relation between an unaccented (clitic) and an accented word (content word).

In general, our analysis of different forms of relative prominence regarding the child speech data not only allows for a better understanding of their own characteristics and role in this type of speech but proves the efficiency of a prosodic organization model that supports such a principle. Additionally, the different degrees of prominence – established in each of the four prosodic constituents – lead the language phenomena linked to prosodically strong and weak parts of utterances to relate differently with them depending on the degree.

## CONCLUSION

In the relationship between hesitations and the relative prominence of the four prosodic constituents analyzed when considering hesitation distribution onto strong and weak positions, and also keeping in mind the large quantity of child speech data analyzed, we present the following findings: (1) hesitation events were more frequently identified at weak positions, that is, more hesitations in non-prominent prosodic positions, in line with the literature; (2) although the analyses of the four prosodic constituents corroborate the literature due to the greater tendency for hesitations in weak positions in the four constituents, the incidence of hesitations in strong positions was also observed, albeit less frequently, and (3) the clitic group was the most frequent constituent to present hesitation events in weak positions, followed by phonological phrase constituents, intonational phrase, and phonological utterances, highlighting differences between the emergence of hesitations and the degree of prominence in each prosodic constituent.

Therefore, we conclude that our analysis of the different forms of relative prominence in child speech not only allows for a better understanding of their own characteristics and their role in this type of speech but proves the efficiency of a prosodic organization model that supports such a principle.

Grounded in linguistic studies, such conclusions can contribute significantly to the field of Speech Therapy, especially for speech assessment/therapy aimed at speech fluency. Firstly, they highlight the fact that in actual communication situations, speech is naturally characterized by elements that break its flow such that continuous flow is rarely observed in spontaneous speech.

Secondly, our conclusions draw attention to the fact that the hesitations occurred in prosodically weak parts of prosodic constituents, providing Speech Therapy with an additional element for consideration during speech fluency assessment/therapy.

Thirdly, our conclusions draw attention to the importance of phonological aspects not only regarding shorter sound units (phonemes and syllables) but longer units as well (clitic group, phonological phrase, intonational phrase, and phonological utterance). This was particularly the case because, as in a previous study^(17)^, hesitations do not emerge randomly but tend to occur in weaker phonological points of speech both in shorter and longer units.

In conclusion, this study limitations for both the *corpus* and the phenomena analyzed: (1) data collected from a single age group – children with an average age of 5.7; (2) context of typical language development; (3) accounting for all types of hesitations in terms of the prosodic constituents without relating the different types of hesitations individually or their relation with prosodic constituents; (4) analysis of only one discourse genre: interview, and (5) disregarding pragmatic/interpersonal roles between interviewer and child during the interviews. Therefore, these limitations should be further addressed in forthcoming studies.

## Footnotes

1 Meaning in English: and the others also had it, didn’t they?

2 Meaning in English: nods his head yes

3 Meaning in English: who were the characters in the story?

4 Meaning in English: long pause – the interviewer waits for an answer

5 Meaning in English: I remember you were the only one in the room who remembered how Portinari died + tell me about it

6 Meaning in English: with the lead in the paint

7 Meaning in English: tell me what ingredients you would put in this cake?

8 Meaning in English: and what did you play together?

9 Meaning in English: basketball, bowling, hot potato

10 Meaning in English: can you think of anything else that you will have at the graduation?

11 Meaning in English: after we finish dancing, we will have some water and leave

12 Meaning in English: what was the drum like?

13 Meaning in English: just put it in your belt and play with two drumsticks

14 Meaning in English: what is this super important thing you want to talk about?

15 Meaning in English: I want to talk about the xylophone

16 Meaning in English: and when they got to the house, what did they do?

17 Meaning in English: the city mouse offered a table with LOTS of food

18 Meaning in English: what instruments do you remember?

19 Meaning in English: those that the little children were playing

20 Meaning in English: and what did you play together?

21 Meaning in English: hot potato

22 Meaning in English: why do we become strong and healthy when we eat moringa?

23 Meaning in English: so we don’t get sick

24 Meaning in English: and then, why did they go there

25 Meaning in English: they ran down

26 Meaning in English: and is the drum also made of rope?

27 Meaning in English: drum is made of the thing below the plate
