# Supplementary material for: Hesitations and relative proeminence in prosodic constituents in children's speech
Source: Codas. 2022 Jan 10;34(2):e20200220. doi: 10.1590/2317-1782/20212020220 (PMC9769412; doi:10.1590/2317-1782/20212020220)
Supplement: Translation

# Translation: Hesitações e proeminência relativa em constituintes prosódicos na fala infantil

**Authors:** Cristyane de Camargo Sampaio Villega, Lourenço Chacon

**Affiliations:** 1 Programa de Pós-Graduação em Estudos Linguísticos, Universidade Estadual Paulista – UNESP - São José do Rio Preto (SP), Brasil.

**Keywords:** Linguagem Infantil, Linguística, Fonoaudiologia, Comunicação, Desenvolvimento da Linguagem, Fala

## Abstract

**Objetivo:**

verificar se as hesitações ocorreriam, preferencialmente, em posições fortes ou fracas de quatro dos constituintes prosódicos: enunciado fonológico, frase entonacional, frase fonológica e grupo clítico.

**Método:**

os dados foram extraídos de um banco composto por 147 situações de entrevistas realizadas com crianças de 5-6 anos de idade. Para análise dos constituintes prosódicos, foi utilizado o princípio da proeminência relativa. A partir desse princípio, foram consideradas como em posição forte as ocorrências hesitativas identificadas em elementos proeminentes na organização de cada um dos constituintes prosódicos e, como em posição fraca, as ocorrências hesitativas identificadas em partes de constituintes que circundam as posições proeminentes. Foram detectadas, pelos juízes, 2.399 ocorrências hesitativas.

**Resultados:**

foram identificadas, respectivamente, em posições fortes e fracas os seguintes totais de hesitações: (1) no enunciado fonológico = 305 (28,37%) e 770 (71,63%); (2) na frase entonacional = 285 (20,67%) e 1094 (79,33%); (3) na frase fonológica = 129 (16,49%) e 653 (83,51%); e (4) no grupo clítico = 154 (15,21%) e 859 (84,79%).

**Conclusão:**

embora as ocorrências hesitativas tenham sido identificadas em posições fortes em todos os constituintes prosódicos analisados, houve prevalência pela posição fraca. Esse resultado corrobora estudos que afirmam que as hesitações ocorreriam em porções prosodicamente não nucleares. Além dessa confirmação, os resultados reforçam a eficácia do modelo da fonologia prosódica em relação ao princípio da proeminência relativa.

## INTRODUÇÃO

Na literatura, os estudos que tiveram como papel central as hesitações têm partido de diferentes perspectivas teórico-metodológicas. Frequentemente, nesses estudos, hesitações são vinculadas à disfluência, a fim de se buscar o que a diferenciaria de sua contraparte da fala: a fluência. Na busca de padrões de (dis)fluência, investiga-se a relação entre a normalidade e a patologia^(1-7)^, situação em que as hesitações são consideradas como marcas de disfluências comuns, ou seja, marcas que rompem o fluxo da fala, mas tidas como comuns a todos os falantes^(1-7)^.

Diferentemente dessa abordagem, há trabalhos que partem da premissa de que a fluência é algo abstrato e ideal, já que, em situações reais de comunicação, a fala se caracteriza pela presença de elementos que rompem o seu fluxo. Partem, ainda, da premissa de que o aspecto verdadeiramente contínuo desse fluxo raramente é alcançado na fala espontânea. Assim, encontram-se trabalhos que investigaram o papel das hesitações: (1) no planejamento cognitivo da fala^(8-10)^; (2) nos aspectos interacionais da formulação do texto falado^(11, 12)^; e (3) nos aspectos subjetivos e discursivos da fala^(13-19)^.

Dentre os trabalhos que investigaram os papeis da hesitação, sem ter como principal preocupação a relação fluência/disfluência, há estudos que se preocuparam com a relação entre hesitações e prosódia^(20-25)^.

Destaque-se que o termo *prosódia* abrange variados fenômenos, como “[...] parâmetros de altura, intensidade, duração, pausa, velocidade de fala, bem como o estudo dos sistemas de tom, entonação, acento e ritmo das línguas naturais”^(26)^. Desse modo, existiriam dois polos de interesse nos estudos prosódicos: o “mais fonético” e o “mais fonológico”^(27)^.

Na literatura, encontram-se tanto trabalhos que relacionaram as hesitações com aspectos prosódicos tidos como fonéticos^(24, 25)^, quanto trabalhos que fizeram essa relação de um ponto de vista fonológico.

Deste último ponto de vista, tem sido investigada a fala da criança^(13, 20)^, a fala do adulto^(22)^ e a comparação entre a fala do adulto e a da criança^(23)^. Embora analisados sujeitos com faixas etárias diferentes, esses estudos chegaram a conclusão semelhante: porções mais fluentes do enunciado – aquelas sem marcas de hesitação – estiveram presentes em unidades portadoras de acentos, ou em núcleos de grupos entonacionais. Já as porções disfluentes – marcadas por hesitações – ocorreram em partes periféricas e fronteiriças, anteriores aos núcleos, ou seja, “[...] antes da cabeça de constituinte prosódico”^(22)^.

Na fala de crianças, essa distribuição das hesitações foi interpretada como “[...] tentativas infrutíferas de segmentação em blocos prosódicos”^(13)^. Essa distribuição entre porções fluentes e disfluentes na fala das crianças foi também interpretada como “[...] ajuste prosódico, sobretudo de cunho rítmico, na elaboração de enunciados mais longos e mais complexos discursivamente”^(22)^.

Na fala do adulto, ao serem analisadas as marcas hesitativas, verificou-se que a distribuição das hesitações não foi prosodicamente aleatória, já que elas se apresentaram em partes prosodicamente fracas dos enunciados^(22)^.

Já na comparação entre a fala do adulto e a fala da criança, chegou-se à conclusão de que, embora as hesitações tenham se manifestado de modo diferente no adulto e na criança, sua distribuição não foi aleatória do ponto de vista de sua relação com constituintes prosódicos. Assim como apontado nos trabalhos anteriores, as hesitações ocorreram, frequentemente, em sílabas átonas do início de enunciados e nunca em sílabas portadoras de acento. Verificou-se, também, que as hesitações mostraram a “[...] sensibilidade da criança às fronteiras prosódicas ao mesmo tempo em que se revelam nas elaborações mais complexas da produção de enunciados mais longos e da entrada do sujeito nas construções semântico-pragmático-discursivas mais elaboradas”^(23)^.

Como se pode verificar nesses trabalhos, embora tenham sido levantadas questões bastante importantes – como o apontamento de haver interação entre hesitação e prosódia e de haver uma distribuição não aleatória das hesitações, se vistas em sua relação com a prosódia –, detectam-se neles, porém, limitações. Por exemplo, no primeiro trabalho^(13)^, não são especificados nem a quantidade de crianças analisadas, nem os constituintes prosódicos analisados. No segundo trabalho^(22)^, embora tenham sido priorizados três constituintes de diferentes dimensões prosódicas, a investigação foi feita com um único sujeito adulto e, nele, foram analisadas apenas as hesitações que, linguisticamente, se mostraram sob forma de *repetição hesitativa* e de *alongamento hesitativo*. Similarmente, o terceiro trabalho^(23)^ ateve-se, uma vez mais, a marcas hesitativas específicas (*repetição* e *alongamento*) na comparação entre a fala de apenas uma criança e de um único adulto.

Dadas essas limitações, o objetivo do presente trabalho é confirmar se as hesitações ocorreriam, de fato, em posições não proeminentes de quatro dos constituintes que compõem a hierarquia prosódica: *enunciado fonológico*, *frase entonacional*, *frase fonológica* e *grupo clítico*. A escolha desses quatro constituintes se justifica pelo fato de se tratar daqueles que mostram, na continuidade da fala, os diferentes níveis de sua organização acima da palavra, ou seja, os diferentes graus de combinações entre palavras na produção do enunciado.

Pretende-se, com esse objetivo, ampliar o alcance dos trabalhos acima expostos, já que, diferentemente deles, a análise privilegiará: (1) grande número de produções de fala de crianças, especificamente 147 situações de entrevistas; (2) ocorrências de todas as marcas hesitativas que se mostraram nos dados analisados: pausa silenciosa, pausa preenchida, alongamento hesitativo, corte brusco, repetição hesitativa, gaguejamento e falso início; e (3) quatro dos constituintes que compõem a hierarquia prosódica.

Como contribuição, acredita-se que a presente investigação poderá expandir a compreensão, no plano teórico, das relações entre hesitação e prosódia em seu funcionamento típico na fala infantil. Como desdobramento do avanço teórico, espera-se que esta pesquisa contribua com uma melhor formação de terapeutas da linguagem, colaborando, assim, para o desenvolvimento de novos olhares para a avaliação e para a terapia da fala infantil.

## MÉTODO

### Procedimentos éticos

Este estudo foi aprovado pelo Comitê de Ética em Pesquisa da Faculdade de Filosofia e Ciências, Universidade Estadual Paulista “Júlio de Mesquita Filho” – UNESP – Marília, sob o número 0132/2010.

Para a realização da coleta de dados e elaboração da pesquisa, foram incluídas as crianças cujos responsáveis assinaram um Termo de Consentimento Livre e Esclarecido. Além da assinatura desse termo, todas as crianças passaram por triagem fonoaudiológica. Aquelas que apresentaram algum padrão desviante após essa triagem, foram excluídas da amostra e encaminhadas para avaliação.

### Amostra

O material analisado foi retirado do banco de dados *Apropriação do conhecimento da linguagem infantil* (ACoLI), que reúne amostras de fala de 24 crianças com média de idade de 5,7 anos. No período de coleta dos dados (ano de 2011), tais crianças frequentavam, em período integral, o nível II de uma escola municipal de educação infantil (EMEI) do interior paulista.

A formação do banco ACoLI, durante o ano de 2011, baseou-se em dez oficinas pedagógicas realizadas da seguinte maneira: (i) mensalmente foi registrado, em sessões videogravadas por duas documentadoras, o desenvolvimento de tais oficinas em sala de aula pela professora responsável; (ii) após uma semana do registro dessas oficinas, cada criança da sala de aula foi entrevistada, individualmente, por uma das documentadoras; (iii) essas entrevistas foram integralmente registradas (em áudio e em vídeo) no interior de uma cabine acústica instalada na instituição de coleta, com uso de equipamentos de alta fidelidade.

Posteriormente, as gravações de cada situação de entrevista foram transcritas por integrantes do Grupo de Pesquisa “Estudos sobre a Linguagem” (GPEL/CNPq), treinados especificamente para a realização das transcrições, num total de 147. Para organizar a exposição dos dados, foi adotada a seguinte identificação das crianças: S01, S02... S23, S24. Para as documentadoras, foi adotada a identificação D01 e D02. Por fim, as situações de entrevista foram enumeradas de 1 a 10 de acordo com a ordem cronológica de realização de cada oficina pedagógica.

### Critérios de análise

Como marcas linguísticas das hesitações, foram consideradas: pausa silenciosa (*+*), pausa preenchida (*éh*, *áh*, *hum*), alongamento hesitativo (**::**), repetição hesitativa (*palavras repetidas*), corte brusco (/), gaguejamento (*parte de palavras repetidas*) e falso início (*parte inicial de enunciado abandonado*)^(11, 15)^

Foram selecionados os quatro constituintes cujas características prosódicas se mostram acima do nível da palavra na Fonologia Prosódica^(28)^. Conforme antecipado, essa seleção se justifica pelo fato de que eles envolvem não apenas características estruturais das palavras, mas, sobretudo, a relação entre palavras – continuidade fortemente relacionada à fluência na produção dos enunciados. Trata-se dos constituintes *Enunciado Fonológico*, *Frase Entonacional*, *Frase Fonológica* e *Grupo Clítico*. Destaque-se que, no modelo da Fonologia Prosódica^(28)^, esses quatro constituintes se mostram hierarquicamente organizados, de tal modo que (em sentido descendente) um Enunciado Fonológico é composto de uma ou mais Frases Entonacionais, que, por sua vez, se compõem de uma ou mais Frases Fonológicas, as quais se compõem de um ou mais Grupos Clíticos^(28, 29)^.

No interior de cada um dos quatro constituintes, as ocorrências de hesitações foram classificadas conforme ocorressem em suas posições prosodicamente fortes e fracas. Para essa classificação, foi levado em consideração o *Princípio 4* ^(28)^ que rege a Fonologia Prosódica. Nesse princípio, é descrita a relação de proeminência relativa estabelecida entre suas partes, de tal modo que, em um dado constituinte prosódico, um elemento tem **valor forte** e os demais elementos que o circundam o **valor fraco**. Desse modo, as posições foram definidas da seguinte maneira:

- **posição forte** – aquela que corresponde ao elemento em destaque na organização de cada um dos níveis da hierarquia prosódica investigados:
  - a *frase entonacional* proeminente na constituição de um *enunciado fonológico*;
  - a *frase fonológica* proeminente na constituição de uma *frase entonacional*;
  - o *grupo clítico* proeminente na constituição de uma *frase fonológica*;
  - a *palavra fonológica* na constituição de um *grupo clítico.*
- **posição fraca** – aquela que corresponde a constituintes e/ou a partes de constituintes que circundam as posições fortes destacadas acima.

### Quantificação dos enunciados

No conjunto das 147 situações de entrevistas foi computada uma média de 260,7 segundos de duração em cada entrevista e uma média de 33,82 enunciados produzidos pelas crianças, também em cada entrevista. No entanto, foram excluídos aqueles em que não houve material linguístico, ou seja, foram excluídos os enunciados em que a criança respondeu com gestos (Exemplo 01) ou, ainda, os momentos em que houve silêncio e/ou o interlocutor ficou à espera de uma resposta (Exemplo 02):

**Exemplo 01:** (situação de entrevista 01)

**D02** eh os outros também tinham né?

**S01** ((faz gesto de sim com a cabeça))

**Exemplo 02:** (situação de entrevista 02)

**D01** quem eram as personagens da história?

**S02** ((longa pausa - o interlocutor fica à espera de uma resposta))

Dessa forma, foi computado um total de 3.674 enunciados que apresentaram material linguístico. Desse total de enunciados, foram identificados, pelos juízes, aqueles sem nenhuma ocorrência de hesitações e aqueles com ocorrências de hesitação em algum ponto da cadeia linguística, distribuídos na Tabela 1, a seguir:

**Tabela 1:** Distribuição dos enunciados com e sem ocorrências hesitativas

**ENUNCIADOS**	**N. (%)**
**Sem ocorrências hesitativas**	1.880 (53,05%)
**Com ocorrências hesitativas**	1.674 (46,95%)
**Total**	**3.674 (100%)**

**Legenda:** N ***=*** número total de enunciados

Segue-se exemplo de enunciado sem nenhuma ocorrência de hesitação identificada:

**Exemplo 03:** (situação de entrevista 06)

**D01** eu lembro que você foi o único na sala que lembrou como é que o Portinari morreu + conta pra mim

**S04** com o chumbo da tinta

A seguir, apresentamos exemplos de enunciados com ocorrências hesitativas identificadas em algum ponto da cadeia linguística:

**Exemplo 04:** (situação de entrevista 05)

**D02** me conta o que que ia de ingrediente nesse bolo?

**S18** + hum + eu não sei

**Exemplo 05:** (situação de entrevista 09)

**D01** e do que vocês brincaram com elas?

**S13** éh de basquete + de boliche + de:: + de:: ++ de:: ++ batatinha quente

Em 04, verifica-se apenas uma ocorrência de hesitação indiciada por uma marca hesitativa complexa (*+ hum +*), já que nela se combinam duas pausas silenciosas (+) e uma pausa preenchida (*hum*). Já em 05, foram detectadas duas ocorrências: a primeira, mostrada por meio de uma marca hesitativa simples, a pausa preenchida (*éh*); a segunda, mostrada por meio de uma marca hesitativa complexa (*de:: + de:: ++ de:: ++*), em que se combinam repetição hesitativa, alongamento hesitativo e pausa silenciosa.

Assim como nos exemplos 04 e 05, em todas as produções de fala, todos os enunciados em que se detectaram hesitações se mostraram ora com uma única ocorrência hesitativa, como em 04, ora com mais de uma ocorrência hesitativa, como em 05. Dessa forma, em seu conjunto, foi identificado um total de **2.399 ocorrências** de hesitações.

### Análise estatística

Foi realizado tratamento estatístico dos dados com o uso do *software Statistica* (versão 7.0). Foram realizadas análise descritiva e análise inferencial. Também foi utilizado o teste paramétrico *T-test de Student* para variáveis dependentes – na comparação entre ocorrências hesitativas identificadas em posições fortes e em posições fracas em cada um dos quatro constituintes prosódicos. A escolha do teste paramétrico se baseou na verificação da não violação da curva do teste de normalidade, adotando-se o valor de α ≤ 0,05.

## RESULTADOS

Em todas as 147 situações de entrevistas foram identificadas, pelos juízes, hesitações em algum ponto da cadeia linguística e relacionadas aos quatro constituintes prosódicos analisados (enunciado fonológico, frase entonacional, frase fonológica e grupo clítico). Na Tabela 2, encontra-se a distribuição dessas hesitações em posições fortes e fracas dos quatro constituintes prosódicos, bem como sua distribuição estatística:

**Tabela 2:** Análise estatística descritiva e inferencial da distribuição entre posição forte e posição fraca no interior dos quatro constituintes prosódicos

**Constituintes prosódicos**	**Posição forte**	**Posição fraca**	**Teste t de Student**
***Enunciado fonológico*** (*n* = 1.075)	28,37% (305)	71,63% (770)	t = - 4,072 p = 0,0005* df = 22
***Frase entonacional*** (*n* = 1.381)	20,64% (285)	79,36% (1096)	t = - 5,408 p = 0,00002* df = 22
***Frase fonológica*** (*n* = 788)	16,37% (129)	83,63% (659)	t = - 5,444 p = 0,00001* df = 22
***Grupo clítico*** (*n* = 1.013)	15,20% (167)	84,80% (859)	t = - 7,0441 p = 0,0001* df = 22

Teste t para amostras dependentes (p≤0,05)

**Legenda:** *n* ***=*** número total de ocorrências hesitativas em cada nível da hierarquia

* = diferença estatisticamente significativa

Verifica-se, na Tabela 2, que, nos quatro constituintes prosódicos, as hesitações emergiram, preferencialmente, em posições fracas.

Seguem-se exemplos de ocorrências hesitativas identificadas em posições **fortes** e **fracas** nos quatro constituintes analisados. Primeiramente, segue-se exemplo em posição **forte** de enunciado fonológico:

**Exemplo 06:** (situação de entrevista 10)

**D01** você lembra de mais alguma coisa que vai ter na formatura?

**S12** depois que nós parar de dançar + nós vamos beber uma aguinha **e vamos + e vamos + e vamos** embora

No Exemplo 06, o enunciado fonológico é composto por três frases entonacionais: (1) *depois que nós parar de dançar*; (2) *nós vamos beber uma aguinha*; e (3) *e vamos embora*. De acordo com os princípios que regem esse nível da hierarquia, a frase entonacional mais à direita do enunciado é aquela que naturalmente receberá o valor forte, por delimitar o término prosódico e sintático do enunciado. Como se pode observar, a ocorrência hesitativa *e vamos + e vamos + e vamos* – indiciada pelas marcas repetição hesitativa e pausa silenciosa – foi identificada justamente na frase entonacional que corresponde à posição forte do enunciado.

Segue-se exemplo de ocorrência hesitativa identificada em posição **fraca** de enunciado fonológico:

**Exemplo 07:** (situação de entrevista 01)

**D02** como é que era o tambor?

**S15** **+ é::** só colocar na cintu::ra + e tocar com dois pauzinhos

Em 07, o enunciado fonológico compõe-se de duas frases entonacionais: (1) *é só colocar na cintura* e (2) *e tocar com dois pauzinhos.* A ocorrência hesitativa – indiciada pelas marcas pausa silenciosa e alongamento hesitativo (*+ é::*) – foi identificada na primeira frase entonacional. Como ela antecede a frase entonacional que apresenta o acento prosódico, a ocorrência hesitativa se situa na porção prosodicamente fraca do enunciado fonológico.

Segue-se exemplo de ocorrência hesitativa identificada em posição **forte** de frase entonacional:

**Exemplo 08:** (situação de entrevista 01)

**D01** é sobre o que essa coisa super importante que você quer falar?

**S13** eu quero falar do:: ++ xilofone

Em 08, a frase entonacional *eu quero falar do xilofone* é composta por duas frases fonológicas: (1) *eu quero falar*; e (2) *do xilofone*. Como se trata de uma frase entonacional em que não se detectam processos enfáticos em uma de suas frases fonológicas, a ocorrência hesitativa – indiciada pelas marcas alongamento hesitativo e pausa silenciosa (*do:: ++* ) –, foi identificada no interior da segunda frase fonológica, aquela que se apresentou com maior destaque prosódico e, portanto, considerada com valor forte nessa frase entonacional. Esse maior destaque na segunda frase fonológica é reforçado pelo fato de ela corresponder à porção que finaliza um enunciado.

A seguir, mostra-se exemplo de ocorrências hesitativas identificadas em posição **fraca** de frase entonacional.

**Exemplo 09:** (situação de entrevista 08)

**D02** e chegando lá na casa o que que eles fizeram?

**S20** hum:: + o rato da cidade ofereceu + uma:: + mesa com MUIta comida

Em 09, também há uma única frase entonacional, composta por três frases fonológicas: (1) *o rato* *da cidade*; (2) o*fereceu* *uma mesa*; e (3) *com MUIta comida*. Nesse exemplo, foram identificadas duas ocorrências hesitativas (*hum:: +*, indiciada pelas marcas pausa preenchida, alongamento hesitativo e pausa silenciosa) e (*+ uma:: +*, indiciada pela combinação entre pausas silenciosas e alongamento), ambas em frases fonológicas anteriores à frase fonológica proeminente (*com MUIta comida*) da frase entonacional; portanto, incidiram sobre porções identificadas como fracas desse constituinte. Destaca-se que a proeminência prosódica desta última frase fonológica é reforçada pelo próprio fato de haver um elemento transcrito com letras maiúsculas (o que demonstra ênfase na fala).

Depois de mostrados exemplos de ocorrências de hesitação no constituinte frase entonacional, parte-se para a descrição delas no constituinte imediatamente inferior: a frase fonológica. Segue-se exemplo de ocorrência hesitativa identificada em posição **forte** desse constituinte:

**Exemplo 10:** (situação de entrevista 07)

**D01** quais instrumentos que você lembra?

**S14** aquela que as criancinhas ‘tava **to/ + éh tá + to** cando

Em 10, há uma frase entonacional composta de três frases fonológicas: (1) *aquela*; (2) *que as criancinhas*; e (3) *tava tocando*. A ocorrência hesitativa (*to/ + éh tá +*) – mostrada pela combinação entre gaguejamento, pausa silenciosa e pausa preenchida – incide sobre a terceira frase fonológica (*tava tocando*), que é composta, por sua vez, de dois constituintes imediatamente abaixo: os grupos clíticos (1) *tava* e (2) *tocando*. De acordo com as regras de proeminência relativa desse nível da hierarquia prosódica, em línguas como o Português Brasileiro, o grupo clítico mais à direita de uma frase fonológica é sempre forte. Todos os demais grupos clíticos que o acompanham são sempre fracos. Dessa forma, a ocorrência hesitativa, em 10, incide sobre o grupo clítico mais à direita, e, portanto, detectada em posição forte.

A seguir, exemplifica-se ocorrência hesitativa identificada em posição **fraca** de frase fonológica:

**Exemplo 11:** (situação de entrevista 09)

**D01** e do que vocês brincaram com elas?

**S13** de boliche + de:: + de:: ++ de:: ++ batatinha quente

Em 11, a ocorrência hesitativa indiciada pelas marcas repetição hesitativa, alongamento hesitativo e pausa silenciosa (*de:: + de:: ++ de:: ++*) incide sobre a frase fonológica *de batatinha quente*. Essa frase fonológica, por sua vez, se compõe dos grupos clíticos: (1) *de batatinha*; e (2) *quente*. A ocorrência hesitativa encontra-se no grupo clítico à esquerda, aquele com o valor fraco (*de:: + de:: ++ de:: ++ batatinha* ), uma vez que, nesse nível da hierarquia que corresponde à frase fonológica, a porção proeminente está sempre no grupo clítico mais à direita – no caso, correspondendo à palavra *quente*.

Agora, será exemplificada ocorrência hesitativa identificada em posição **forte** de grupo clítico:

**Exemplo 12:** (situação de entrevista 08)

**D02** por que que a gente fica forte e saudável se comer a moringa?

**S08** *pra* ***gen::te:: +*** *não ficar* doente

De acordo com os princípios da Fonologia Prosódica, o constituinte *grupo clítico* é formado por uma palavra de conteúdo e todos os elementos clíticos (palavras monossilábicas não acentuadas, como os artigos e algumas preposições, conjunções e pronomes) a sua volta. Dessa forma, considera-se como posição forte, nesse constituinte, quando a ocorrência hesitativa é identificada na palavra de conteúdo que o compõe. Vê-se, em 12, que o grupo clítico *pra gente* é composto por um elemento clítico *pra* e por uma palavra de conteúdo *gente*. A ocorrência hesitativa indiciada pelas marcas alongamento hesitativo e pausa silenciosa (*gen::te:: +*) incidiu, justamente, na palavra de conteúdo e, portanto, na posição forte desse grupo clítico.

A seguir, exemplifica-se ocorrência hesitativa identificada em posição **fraca** de grupo clítico:

**Exemplo 13:** (situação de entrevista 02)

**D02** e aí o que elas foram fazer lá

**S02** elas correram **na + na** descida

Em 13, a ocorrência hesitativa *na + na* – indiciada pelas marcas repetição hesitativa e pausa silenciosa – incidiu sobre o monossílabo não acentuado do grupo clítico *na descida*. Classifica-se essa ocorrência hesitativa em posição fraca de grupo clítico, pois, de acordo com a regra de proeminência relativa desse constituinte prosódico, é sempre fraco o elemento considerado clítico e sempre forte a palavra de conteúdo que compõe esse constituinte.

Expostos os resultados, bem como a exemplificação de ocorrências hesitativas em relação aos quatro constituintes prosódicos analisados, passa-se, a seguir, à discussão dos resultados.

## DISCUSSÃO

Os resultados mostraram que as hesitações se distribuíram tanto em posição forte quanto em posição fraca em cada um dos quatro constituintes prosódicos analisados. No entanto, verificou-se maior porcentagem de ocorrência para a posição **fraca/não proeminente** em todos os constituintes. Essa diferença mostrou-se, ainda, estatisticamente significativa. Tal prevalência de ocorrências hesitativas em posições fracas corrobora dados da literatura que investigou a relação entre hesitações e prosódia (de um ponto de vista fonológico). De acordo com essa literatura, quando as hesitações ocorrem, elas são identificadas preferencialmente em partes não proeminentes dos constituintes^(13, 22, 23)^. Em um dos estudos^(22)^, observou-se que as hesitações não tendem a ocorrer em trechos proeminentes, mas, sim, em trechos “[...] periféricos e fronteiriços anteriores ao núcleo [porção proeminente]”^(22)^. Em outras palavras, a distribuição das hesitações não foi prosodicamente aleatória, uma vez que seguiu um padrão geral de comportamento prosódico.

Desse modo, os resultados do presente estudo confirmam que as ocorrências hesitativas tendem a incidir sobre posições fracas – ou seja, anteriores à proeminência relativa –, como demonstrado nos exemplos: 07, em enunciado fonológico; 09, em frase entonacional; 11, em frase fonológica; e 13, em grupo clítico.

Embora os resultados do presente estudo tenham confirmado a literatura, chama-se atenção para a seguinte afirmação retirada de um estudo que investigou a relação entre hesitações e constituintes prosódicos na fala infantil: “A rigor, **nunca encontrei**, em dados de [hesitações], casos de repetição hesitativa [...] incidindo sobre as sílabas portadoras de acento frasal [...]”^(23)^ (destaque dos autores do presente estudo).

No entanto, nos dados aqui analisados, foram identificadas situações semelhantes àquelas destacadas a seguir:

**Exemplo 14:** (situação de entrevista 01)

**D01** e o tambor também é feito de corda?

**S02** não + tambor é feito + é fe::ito + com negócio embaixo com pra::to

Nesse Exemplo 14, a ocorrência hesitativa incide sobre a frase fonológica *é feito*, composta por dois grupos clíticos (1) *é* e (2) *feito*. Nessa ocorrência, foi identificada a repetição hesitativa de toda a estrutura; portanto, também de sua porção forte: o grupo clítico *feito*. Diferentemente do que foi apontado pela literatura^(23)^, ocorrências como a do Exemplo 14 mostram que as repetições hesitativas, embora em menor grau, podem incidir também sobre o elemento proeminente, ou seja, aquele portador do acento prosódico.

Consequentemente, embora as ocorrências de hesitações em posição fraca tenham prevalecido em todos os constituintes analisados, também se encontra, nos dados, uma parcela de ocorrências hesitativas identificadas em posição forte. Com efeito, diferentemente do que é destacado em trabalhos que investigam relações entre hesitação e prosódia^(11, 22, 23)^, além do Exemplo 14, que exemplifica a posição forte em nível de frase fonológica, também foram verificadas hesitações em partes fortes dos demais constituintes prosódicos, como se pôde observar nos exemplos: 06, em enunciado fonológico; 08, em frase entonacional; e 12, em grupo clítico. Pode-se, pois, pensar que não apenas o valor fraco, em si, provocaria a emergência de hesitações, mas, ainda, que, embora em menor percentual, essa emergência poderia decorrer (também) da relação de proeminência entre porções de um mesmo constituinte – o que explicaria sua presença (também) em porções prosodicamente fortes.

Outro resultado observado nos dados mostra que, dentre as ocorrências hesitativas que incidiram em posição fraca, o grupo clítico foi o constituinte que mais esteve envolvido, percentualmente, nessa posição. O clítico fonológico coincide com a categoria que um estudo^(11)^, ao classificar os tipos de palavras sobre os quais incidem hesitações, chama de itens funcionais – tais como os artigos, as preposições e as conjunções. Observe-se que, embora esse estudo tenha sido desenvolvido com metodologia diferente daquela que orienta a presente pesquisa (ao não relacionar hesitações aos constituintes prosódicos fonológicos) e, ainda, tenha sido desenvolvido com população diferente (já que analisou a fala de adultos), os resultados aqui apresentados convergem com aqueles relatados por esse estudo, o qual relata que 50% das hesitações encontradas na investigação da fala de adultos envolveram os itens funcionais.

Em outro estudo^(30)^, com resultado semelhante, os autores afirmam que “[...] pontos de grande possibilidade de escolha fazem com que o falante tenha dificuldade em selecionar a palavra seguinte no fluxo da fala”^(30)^. Para os autores, as palavras funcionais representam uma dificuldade menor de formulação do que palavras de conteúdo. Deve-se, pois, pensar que, no jogo de proeminência relativa característico de cada nível da hierarquia prosódica, o mais significativo para a emergência de hesitações seja o grupo clítico pelo fato de esse jogo envolver não apenas diferentes graus de proeminência (como ocorre nos constituintes superiores ao do grupo clítico), mas a própria ausência/presença de proeminência, já que se trata da relação entre uma palavra desprovida de acento (clítico) e uma palavra provida dele (palavra de conteúdo).

De modo geral, a análise das diferentes formas de proeminência relativa, aqui apresentada em dados de fala infantil, permite, além de compreender melhor suas próprias características e seu papel nesse tipo de fala, comprovar a eficácia de um modelo de organização prosódica que se sustenta nesse princípio. Como se verifica, os diferentes graus da proeminência – estabelecida em cada um dos quatro constituintes prosódicos – fazem com que os fenômenos da língua vinculados a porções prosodicamente fortes e fracas de enunciados relacionem-se diferentemente com elas, a depender desses graus.

## CONCLUSÃO

Na relação entre hesitações e proeminência relativa dos quatro constituintes prosódicos analisados, no que diz respeito à distribuição das hesitações em posições fortes e fracas e levando-se em consideração a grande quantidade de dados de fala infantil analisados na presente investigação, verifica-se que: (1) houve preferência pela ocorrência de hesitações identificadas em posição fraca, ou seja, ocorreram mais hesitações em porções **não portadoras de proeminência prosódica**, corroborando a literatura; (2) embora as análises dos quatro constituintes prosódicos confirmem a literatura, pela preferência de hesitações em posição fraca dos quatro constituintes, contrariando a mesma literatura, também há, em menor quantidade, hesitações que incidem sobre posições fortes dos constituintes analisados; e (3) das ocorrências hesitativas que incidiram sobre posições fracas, o grupo clítico foi o constituinte que mais esteve envolvido, percentualmente, nessa posição, seguido dos constituintes frase fonológica, frase entonacional e enunciado fonológico, demonstrando haver diferenças entre a emergência de hesitações e o grau de proeminência em cada um dos constituintes prosódicos.

Desse modo, constata-se que a análise aqui realizada das diferentes formas de proeminência relativa na fala infantil permite, além de compreender melhor suas próprias características e seu papel nesse tipo de fala, comprovar a eficácia de um modelo de organização prosódica que se sustenta nesse princípio.

Tais conclusões, embasadas em estudos linguísticos, podem trazer contribuições valiosas para o campo da Fonoaudiologia, principalmente quanto à avaliação/terapia fonoaudiológica voltada à fluência de fala. Em primeiro lugar, chama-se atenção para o fato de que a fala, em situações reais de comunicação, é naturalmente caracterizada pela presença de elementos que rompem o seu fluxo. Sendo assim, o fluxo contínuo da fala é raramente alcançado na fala espontânea.

Em segundo lugar, as conclusões a que chegou a investigação relatada no presente estudo chamam a atenção para o fato de as hesitações ocorrerem em porções prosodicamente fracas dos constituintes prosódicos, o que traz à Fonoaudiologia mais um elemento a ser levado em consideração durante avaliação/terapia voltada à fluência de fala.

Em terceiro lugar, as conclusões chamam a atenção para a importância dos aspectos fonológicos não apenas quanto às unidades sonoras menores da fala (fonemas e sílabas), mas também para suas unidades maiores (grupo clítico, frase fonológica, frase entonacional e enunciado fonológico). Especialmente porque, assim como se viu em trabalho relatado^(17)^, as hesitações não se mostram de forma aleatória, já que tendem a ocorrer em pontos fonológicos mais fracos da fala tanto em suas unidades menores, quanto em suas unidades maiores.

Por fim, verificam-se, no presente estudo, limitações quanto ao *corpus* e quanto aos fenômenos analisados: (1) foram coletados dados de uma única faixa etária – ou seja, crianças com média de 5,7 anos de idade; (2) em contexto de desenvolvimento típico de linguagem; (3) foi considerada a totalidade de todos os tipos de hesitações em relação aos constituintes prosódicos, sem, portanto, relacionar os diferentes tipos de hesitações isolados e sua relação com os constituintes prosódicos; (4) foi analisado um único gênero discursivo: entrevista; e (5) não foram considerados os papeis pragmáticos/interpessoais entre documentadora e criança durante as entrevistas. Tais limitações devem, portanto, constituir-se em objeto de análise em investigações futuras.
